# Temperature effect on water dynamics in tetramer phosphofructokinase matrix and the super-arrhenius respiration rate

**DOI:** 10.1038/s41598-020-79271-5

**Published:** 2021-01-11

**Authors:** Hsiao-Ching Yang, Yung-Chi Ge, Kuan-Hsuan Su, Chia-Cheng Chang, King-Chuen Lin, Vincenzo Aquilanti, Toshio Kasai

**Affiliations:** 1 Department of Chemistry, Fu Jen Catholic University, New Taipei City, 24205 Taiwan; 2 Department of Chemistry, National Taiwan University, Taipei, 10617 Taiwan; 3 Institute of Atomic and Molecular Sciences, Academia Sinica, Taipei, 10617 Taiwan; 4 Dipartimento di Chimica, Biologia e Biotecnologie, Università di Perugia, 06123 Perugia, Italy; 5 Institute of Scientific and Industrial Research, Osaka University, Ibaraki, Osaka 567-0047 Japan; 6 Istituto di Struttura della Materia, Consiglio Nazionale delle Ricerche, 00133 Rome, Italy

**Keywords:** Chemistry, Enzymes

## Abstract

Advances in understanding the temperature effect on water dynamics in cellular respiration are important for the modeling of integrated energy processes and metabolic rates. For more than half a century, experimental studies have contributed to the understanding of the catalytic role of water in respiration combustion, yet the detailed water dynamics remains elusive. We combine a super-Arrhenius model that links the temperature-dependent exponential growth rate of a population of plant cells to respiration, and an experiment on isotope labeled ^18^O_2_ uptake to H_2_^18^O transport role and to a rate-limiting step of cellular respiration. We use Phosphofructokinase (PFK-1) as a prototype because this enzyme is known to be a pacemaker (a rate-limiting enzyme) in the glycolysis process of respiration. The characterization shows that PFK-1 water matrix dynamics are crucial for examining how respiration (PFK-1 tetramer complex breathing) rates respond to temperature change through a water and nano-channel network created by the enzyme folding surfaces, at both short and long (evolutionary) timescales. We not only reveal the nano-channel water network of PFK-1 tetramer hydration topography but also clarify how temperature drives the underlying respiration rates by mapping the channels of water diffusion with distinct dynamics in space and time. The results show that the PFK-1 assembly tetramer possesses a sustainable capacity in the regulation of the water network toward metabolic rates. The implications and limitations of the reciprocal-activation–reciprocal-temperature relationship for interpreting PFK-1 tetramer mechanisms are briefly discussed.

## Introduction

Cellular respiration is perhaps the most important biochemical reaction by which life forms obtain energy to harvest and store in molecules, such as ATP, to be further utilized^1–7^. Usually, the overall reaction of respiration to turn glucose and oxygen into carbon dioxide and water is expressed by Eq. (1)^8^. Our recent mass spectroscopic study to accurately determine the total rate constant of the respiration reaction of Camellia Japonica leaves has confirmed the exact 1:1 correspondence between O_2_ consumption and CO_2_ emission suggested by Eq. (1)^8^.

1 $$ {\text{C}}_{{6}} {\text{H}}_{{{12}}} {\text{O}}_{{6}} + {\text{ 6O}}_{{2}} \to {\text{6CO}}_{{2}} + {\text{ 6H}}_{{2}} {\text{O}} $$

However, mass spectroscopic measurement using a heavy ^18^O_2_ marker revealed that H_2_O indispensably is involved in the reaction; thus, Eq. (1) has to be revised into the form shown in Eq. (2)^8–11^. At present, we do not exactly understand the explicit role of water as the mechanism in Eq. (2); i.e., the transport of water can play a critical role as an “assistant” in systematic enzyme catalysis.

2 $$ {\text{C}}_{{6}} {\text{H}}_{{{12}}} {\text{O}}_{{6}} + {\text{ 6H}}_{{2}} {\text{O }} + { 6}^{{{18}}} {\text{O}}_{{2}} \to {\text{6CO}}_{{2}} + {\text{12H}}_{{2}}^{{{18}}} {\text{O}} $$

**Figure 1:**
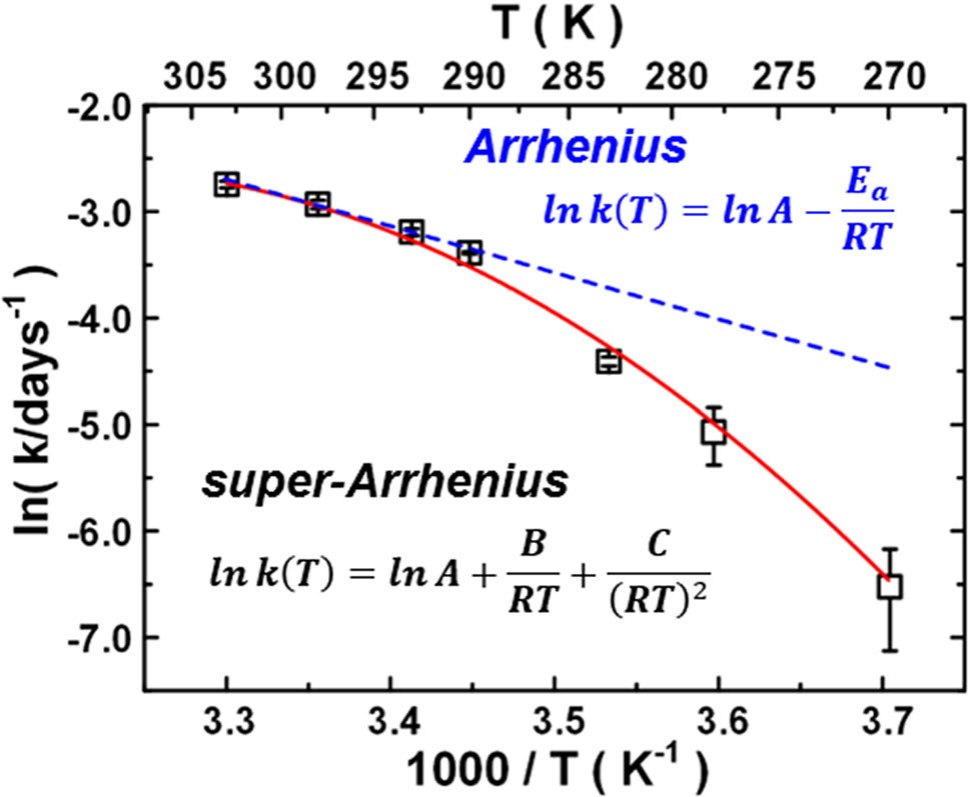
Arrhenius plot of logarithm of reaction rate constants as a function of inverse absolute temperature K. The dashed line in blue indicates the linear Arrhenius plot as a reference. The convex line in red indicates the super-Arrhenius plot as a reference^9^.

We carried out measurements to obtain the temperature dependence of the respiration rate^9^, as revealed in Fig. 1. It clearly indicates non-Arrhenius behavior as the nonlinear function of the natural logarithm of the rate coefficient vs. reciprocal of the absolute temperature (1/*T*)^12^, the so-called super-Arrhenius behavior (the “convex” type deviation), which is (Fig. 1). *E*_a_ is no more than a constant of activation energy but decreases with increasing temperature. By assuming that the activation energy varies with temperature *E*_a_(*T*), we were able to obtain its physical meaning, that the super-Arrhenius behavior would be related to the diffusion kinetics term^9,13^. Therefore, it is worthwhile to investigate how this diffusion kinetics term works at the molecular level^14–16^. There has been an increasing number of studies measuring the temperature dependence of enzyme reactions, showing super- Arrhenius behaviors^12,17–23^. Although theoretical model studies have been advanced for the interpretation of such super-Arrhenius behaviors^12,17,18,20^, space remains for more specific interpretations, such as enzyme complex dynamics in conjunction with a temperature-dependent structural reorganizations and reaction processes^12^. The diffusion in supercooled liquids and glasses have also been observed from super-Arrhenius plots^17,18,20^, which have yielded to detailed analysis with temporal relaxation of supercooled liquids approaching the glass transition by multiple steps. In the present paper, we mention the case of plant cell respiration process^5–7,24^ consisting of multiple reactions, where a super-Arrhenius plot (Fig. 1) has been observed^8,9,13^, the elementary steps have not been completed or cannot be unified^12,17,18^. We draw attention to an analogy with water diffusion in metastable fluids taking place in the relevant respiration enzymes. We try to point out that enzyme complex places a severe constraint on the fluid diffusion for the involved chemical steps toward a super-Arrhenius behavior^12^.

### Scheme of respiration reaction

**Figure 2:**
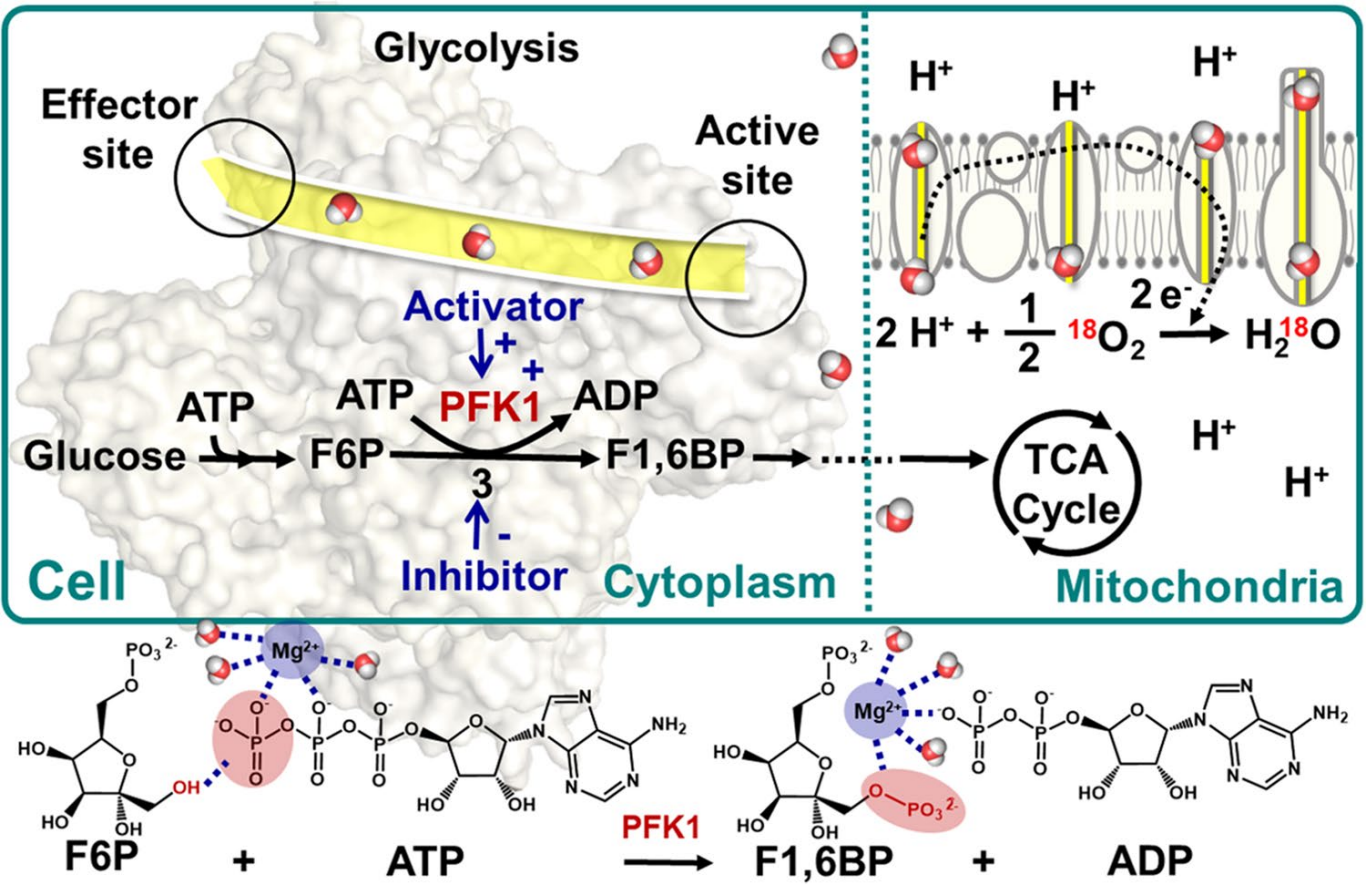
Cellular respiration is a metabolic pathway consisting of two major locations, in the cellular cytoplasm for glycolysis and in mitochondria, which produce ATP, in which the “committed” step of glycolysis at pacemaker PFK-1 activity is modulated by allosteric activators, including ADP, F6P, and phosphate (P_i_), as well as those inactivators of citrate, fatty acids, and ATP. In the whole reaction, water is necessary. There are water channels (shaded yellow) in the protein to regulate the distribution of water.

Cellular respiration involves a long list of metabolic processes^2^, including major portals (Fig. 2) for cytoplasm enzymes towards (1) glycolysis as producers of ATP and mitochondria with (2) the citric acid cycle, and (3) electron transport chain/oxidative phosphorylation^25^; however, the precise roles of each in respiration remain unknown^26^. Glycolysis is the initial step of aerobic respiration, which leads to the generation of pyruvate/lactate, NADH and ATP in anaerobic conditions and finally glucose is oxidized to carbon dioxide^27^. Global metabolic profiling of bacteriostatic antibiotic treatment revealed that the accumulation of metabolites was linked to the buildup of energy metabolites that feed the electron transport chain^28^ and a marked increase in glucose uptake, triggering the activity of phosphofructokinase-1 (PFK-1), a key rate-limiting enzyme of the glycolysis pathway^27,29^. Regarding the “committed” step of glycolysis at PFK-1, it catalyzes the conversion of fructose 6-phosphate (F6P) and adenosine triphosphate (ATP) to fructose 1,6-bisphosphate (F1,6BP) and adenosine diphosphate (ADP), as explicitly shown in Fig. 2. That figure reveals the dominant metabolic regulation, yet it presents a puzzling regulatory mechanism of the allosteric effect between the active and effector sites^14,30^.

Analysis of each step of glycolysis shows that, of those rate-limiting enzymes of the pathway^31,32^, isoforms of PFK-1 may afford the greatest opportunity^33,34^ as targets to explain the complex oligomeric process coupled water dynamics to interpret the super-Arrhenius behavior^20^. In particular, we can go beyond the interpretation that a super-Arrhenius plot can be interpreted as a reciprocal of the diffusion states in between specific configuration ‘entities’ upon temperature, to account for transport behavior to the occurrence of long series of collective events^12^. The super-Arrhenius behavior often manifests because of such as rates of enzymatic catalysis-promoted processes and temperatures, and we can say, under certain conditions, that the microenvironment affects water fluid diffusion in supplying the respiratory chain. First, intracellular water transport through the enzyme might direct the chain and react with oxygen to generate a messenger to sequential reactions. Second, due to its metastable transition for changing the hydrogen-bonding network and diffusible nature, water fluid might act as a messenger because of its diffusion among the specific enzyme matrix, which confers properties to systematic protein conformational change and communicates as a signaling molecule^35^. Third, these bio-water fluids might have hierarchical regulation corroborated enzyme matrix reactions; therefore, the increases in plant cell respiration rate are somehow buffered at temperatures of 285–305 K^9,36^ (Fig. 1) and has been found by recording the oxygen consumption of 283–313 K^23^, to abide super-Arrhenius behavior, possibly to prevent an overshoot of metabolism at high temperatures^37^.

Hence, to examine the contributions of various modes of enzyme regulation of water fluid, type-1 phosphofructokinase (PFK-1) was chosen (Fig. 1), for this enzyme is known to be a pacemaker (a rate-limiting enzyme) in the glycolysis process of respiration^31,32^. In this research, we attempted to develop a more microscopically-detailed interpretation resting on the intricate respiration enzyme of PFK-1 complex structures and employ molecular dynamics simulations to investigate the hydration water diffusive motions through the tetrameric phosphofructokinase conformation dynamics over a physiological temperature range of 270–330 K, where experimental data are available. The PFK-1 tetramer structure places a severe constraint on water diffusion at low temperature by crossing appreciable potential barriers, whereas at high-temperature, water diffusive motions are almost free^1,38,39^. Our model recognizes that as the temperature rises, thermal energies become larger than barrier heights; what is required for a super-Arrhenius plot is that the average energy of water molecules making transitions (in PFK-1 tetramer, diffusive water hops from one local minimum to another through the channels) rises more slowly than the average energy of all hydration water^12^.

## Discussion

### Water influx tetramer PFK-1 enzymatic regulation

Protein-water interactions are crucial factors in PFK-1 conformational reorganization, so molecular dynamics (MD) simulation were carried out to investigate the conformational dynamics of the reactant and the product states in the active site in aqueous solution^40–43^. Remarkably, it is known that PFK-1 undergoes an intricate oligomerization process, shifting among monomers, dimers, tetramers, and more complex oligomeric structures^1,31^. The monomer PFK-1 has one active and one effector site but shows no activity. The equilibrium between PFK-1 dimers and tetramers is directly correlated with activity regulation^26^, in which the activity remains very low up to a dimer level and then increases sharply with a tetramer form in the fully active state^16^. Although evidence for oligomerization between members of PFK-1s to affect the allosteric regulation of glycolysis is increasing, not much is known about the underlying mechanism. PFK-1s have been shown to exist as tetramers via noncovalent interactions, and tetramerization favors the agonist-mediated reaction coupling efficiency^44^, suggesting a role for tetramerization in the internalization properties of coupling effector and active sites^33^.

**Figure 3:**
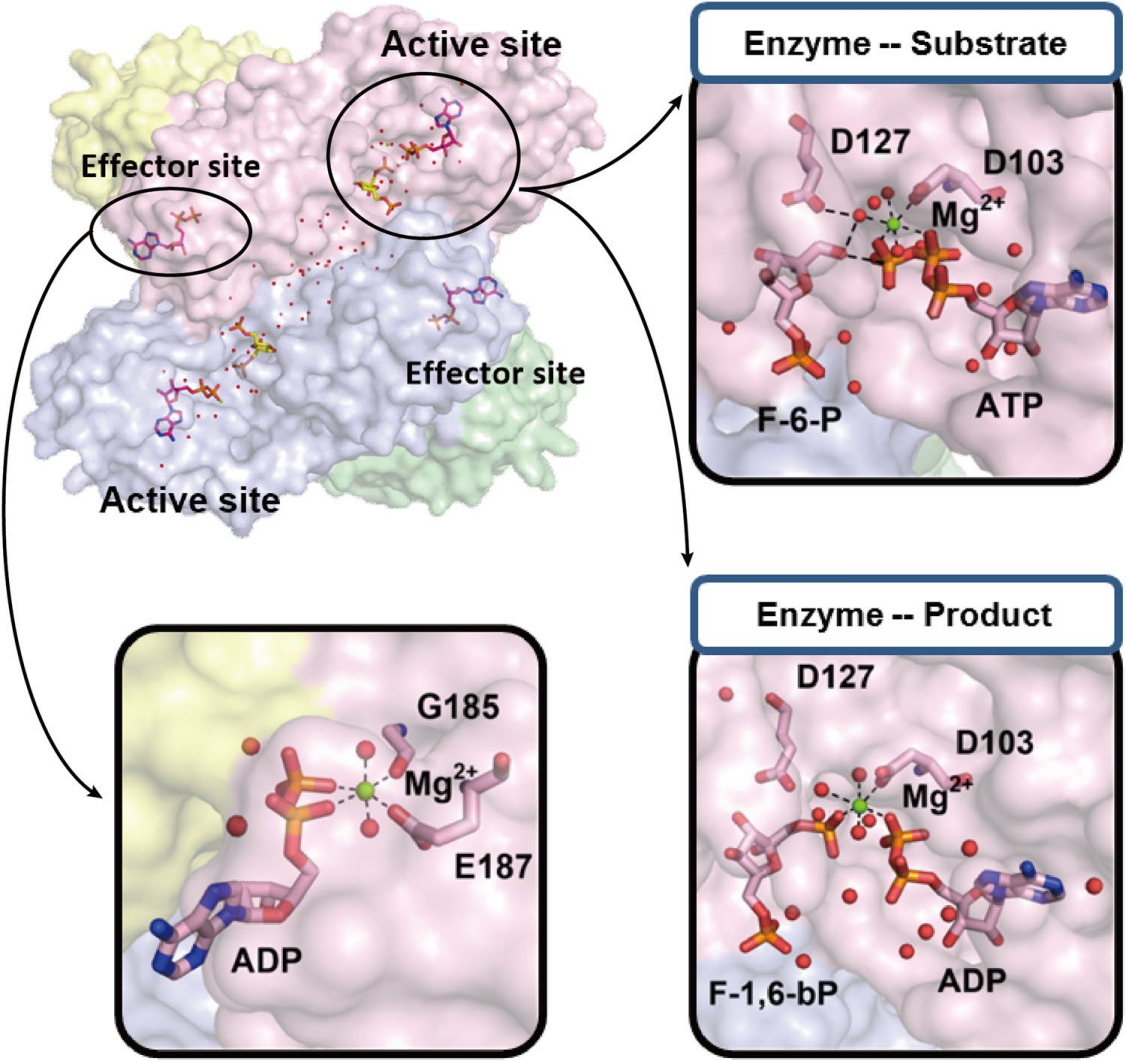
The PFK-1 crystallographic structure (PDB entry 1PFK)^45^ and the enlarged views of an active site occupied as the ‘Enzyme–Substrate’ configuration or as the ‘Enzyme-Product’ configuration. They harbor residues for binding of ADP, F-1, and 6-BP, presented as sticks and colored by atomic type: carbon in pink, nitrogen in blue, oxygen in red, and phosphorus in orange. Magnesium ion (Mg^2+^) and water oxygen are shown as balls colored green and red, respectively. An enlarged view of the ADP bound ‘Effector’ site is shown at the bottom left.

**Figure 4:**
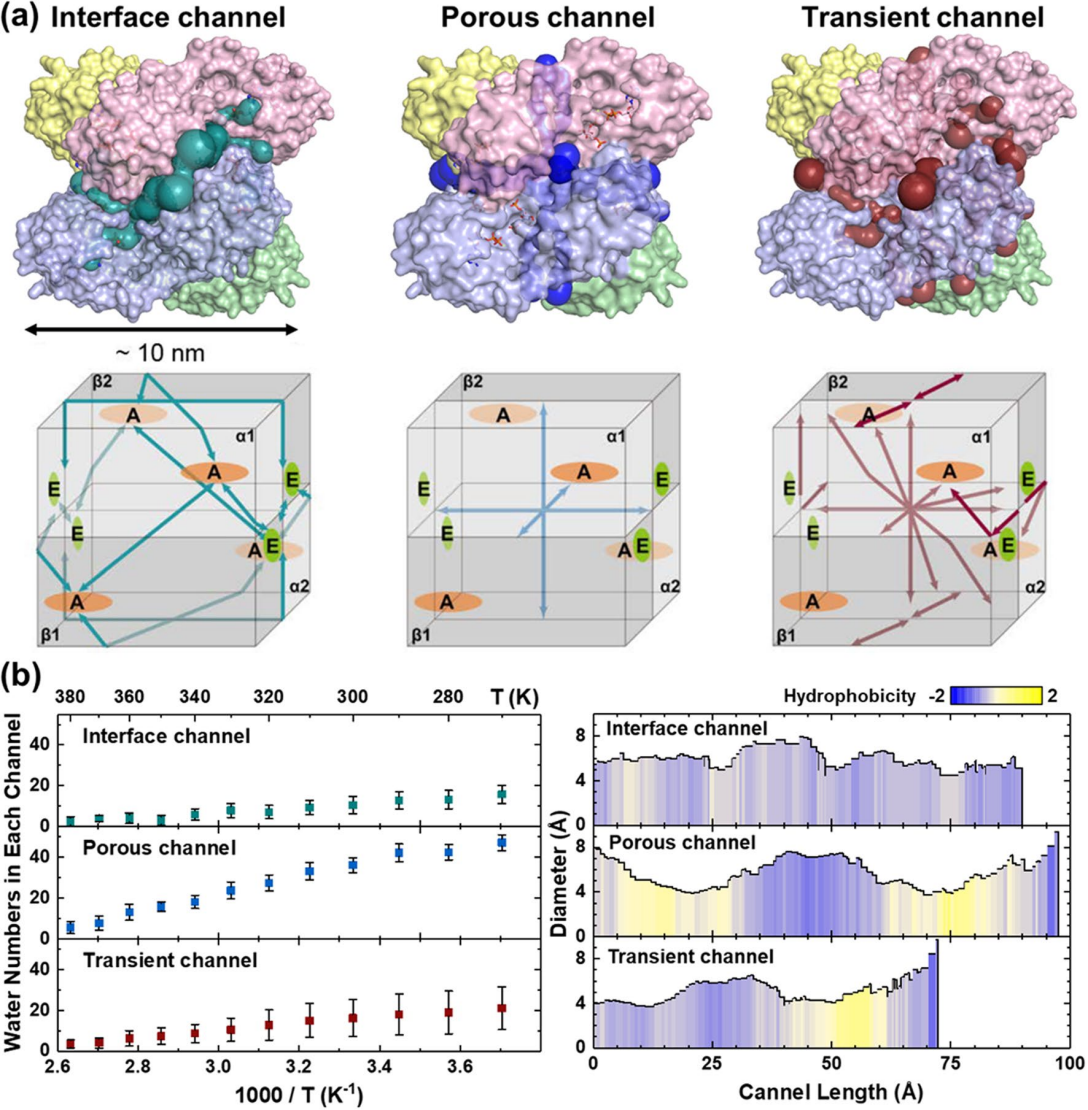
Identification of the site connection channels for the PFK-1 (PDB entry 1PFK)^45^ tetramer matrix^45^, with three categories of (**a**) the interface, the porous channel, and the transient channel. The layout of these channels is mapped in connection with each effector and active site for the tetramer structure by Alphabetic Greek symbols, α_1_, α_2_, β_1_, and β_2_, indicating four PFK-1 monomers. The drawing may not be precise in scale, but it presents the symmetry in the tetramer structure. (**b**) On the left side, numbers of water molecules per channel, with standard deviation within 3 Å of the three different PFK-1 channel centers along the MD structure of 10 frames with a time interval of 100 ps at different temperatures. On the right side, comparison of the channel property with the hydrophobicity of channel residues. The interface channel is evenly distributed, and the porous and transient channels have more hydrophilic residues at the middle and entrance, respectively.

Although a growing number of crystallographic PFK-1 structures has been resolved^33^, it has been possible to identify the interdomain folding feature with findings on the regulatory aspects of PFK-1-water interactions and their relation to the control of metabolism. Figure 3 shows the PFK-1 crystallographic structure (PDB entry 1PFK) of a tetramer assembly^45^, an enlarged view of the ‘Enzyme–Substrate’ site, and one of the ‘Enzyme-Product’ structures at an active site. An enlarged view of an effector site is also shown in the figure. The present analysis has revealed that the magnesium ion (Mg^2+^) coordinates strongly with the relevant water molecules, phosphate ligand and the amino acid of D103. Structural analyses show that the hydration environments are quite different among the active site, effector site and protein interface. Moreover, MD simulation has allowed us to identify the main folding channels and potential site connecting channels in PFK-1 by using the internal structural mining software MOLE*onlin*e^46^, as shown in Fig. 4a. The tetramer assembly folded PFK-1 has revealed the peculiarity of harboring multiple channels in connection with each active and effector site, and the protein interface. With channel search and channel classification, we first tentatively identified the water molecules of the PFK-1 solution into three categories: the internal waters bound in (1) the interface, (2) the porous channel, and (3) the transient channel of PFK-1 enzyme, respectively.

### Features of tetramer PFK-1s with interior site connection water channels

The PFK-1 tetramer assembly is exhibited in Fig. 4a, where each monomer (α or β) has an effector and an active site, the pair-oriented monomer of α and β forms a dimer, and then follows a complex tetramer. The interface channel connects between either an active site (designated as A in the figure) or an effector site (designated as E), referred to as A–A or E–E, respectively. Detailed analyses were conducted on the interface channel (cleft) with PFK-1 tetramer crystallography structures, involving residues crossing two monomers^1,38^. Mutation of the interface amino residues indeed affects PFK-1 activities. Moreover, we find that these PFK-1 monomer complexed interfaces can form multiple different types of interior cavities and/or channels, such as porous and transient channels, for the accommodation of substrate and water molecules. The porous channel connects any active site (or an effector site) and the outside of the enzyme (the bulk water area). The layout of such water channels is schematically drawn for convenience in the bottom panels in Fig. 4a, where the connections to each of the effector and active sites of the PFK-1 tetramer structure are indicated by Greek alphabet symbols, α_1_, α_2_, β_1_, and β_2_, and the interconnections are given with arrows. The drawing is not precise in scale, but it reflects the mutual symmetry within the tetramer enzyme. To be notice of the transient channel is not stable in time, in contrast to the other two channels. However, we can identify the transient channel as the rest of the water channels of the enzyme, which are clearly different from either the interface channel or the porous channel. Each PFK-1 monomer has an active site and an effector site, and these sites are connected by channels in the tetramer complex. In the right side of Fig. 4b, we use MOLE*online*^46^ to calculate and analyze the channels for the crystal structure (PDB entry 1PFK). The interface, porous and transient channel are about 90, 98 and 72 Å long and have bottlenecks of about 4.5, 3.7 and 3.7 Å wide, respectively. The interface channel is rather hydrophilic, for its hydrophobicity is − 0.79 to − 0.08. By comparison, the hydropathy values of the porous and transient channels range from − 1.07 to 0.80 and from − 1.07 to 0.63, respectively, suggesting that these channels are more variable than the interface channel. In terms of time, the transient channel changes more significantly than does the porous channel. In addition, these channels have different abilities to restrict water. The interface channel traps water with hydrophilic amino acids, whereas the porous channel space uses candy-shaped spaces to restrict water movement. Compared with the previous two, the transient channel has a very unstable water volume (note the larger error bar on the left side of Fig. 4b). These reasons make the function of the transient channel in regulating water diffusion very different from those of the other two.

Nevertheless, understanding for the PFK-1 three types of channels their trapped water motions gives specific support to the kinetic models, whose validity should be examined by further experiments and detailed simulations^12^. The present study does specifically invoke these channel water diffusive effects associated with the super-Arrhenius behavior. Such as for uptake of the protons required in water formation on reduction of O_2_ and uptake and release of the protons pumped across the membrane need the restricted channel waters for the proton transfer pathways. Control by temperature that the channels make a close and open form by the amino acid to influence the water-protein interactions in the channel. The channel water diffusion may make a key role in controlling the respiration reaction for the proton coupled electron transfer^26,47–49^ and the O_2_ reduction. It has been found that the protons released need requires a proton-transferring pathway (a “proton well”) in the relevant protein. Due to this, the electrical potential difference is created entirely, or nearly so, by transmembrane proton transfer across the “well”, involving amino acid residues, two of which have been as carboxylates, and perhaps water molecules^26^.

For a quantitative description of hydration water behavior coupled with the PFK-1 enzyme, we performed temperature-dependent MD simulations with explicit solvent water molecules to analyze the water-coupled PFK-1 dynamics. Each channel holds water molecules in a constricted channel diameter and length to permit the trapped water motion to connect between each effector and active site for the interface, and the porous channels and the transient channel function to link the channel water with the bulk area outside of the enzyme. The average numbers of water molecules in each channel are 11 ± 4 for the interface channel, 17 ± 3 for the porous channel, and 15 ± 9 for the transient channel, respectively. The standard deviation of the number is 270–340 over time, on average. Figure 4b displays the number of water molecules in each channel as a function of temperature in units of K and also its reciprocal in units of K^−1^, obtained by MD simulation. Three channel diameters as a function of channel length are also displayed in Fig. 4b, where the channel length is measured from the center of the enzyme. The broad widths along the plots indicate a time-dependent distribution of water in each channel. The channel widths are barely wide enough for a few water molecules to move around under the interaction with the channel wall. Thus, it is expected that the trapped channel water molecules are exchanged dynamically with the PFK-1 enzyme surface water and that the penetration of water molecules into channels significantly varies with temperature. However, the space inside each channel permits the trapped water to move and interconnects each effector and active site at the end of the channel.

### Channel water influx temperature-dependent diffusion kinetics

**Figure 5:**
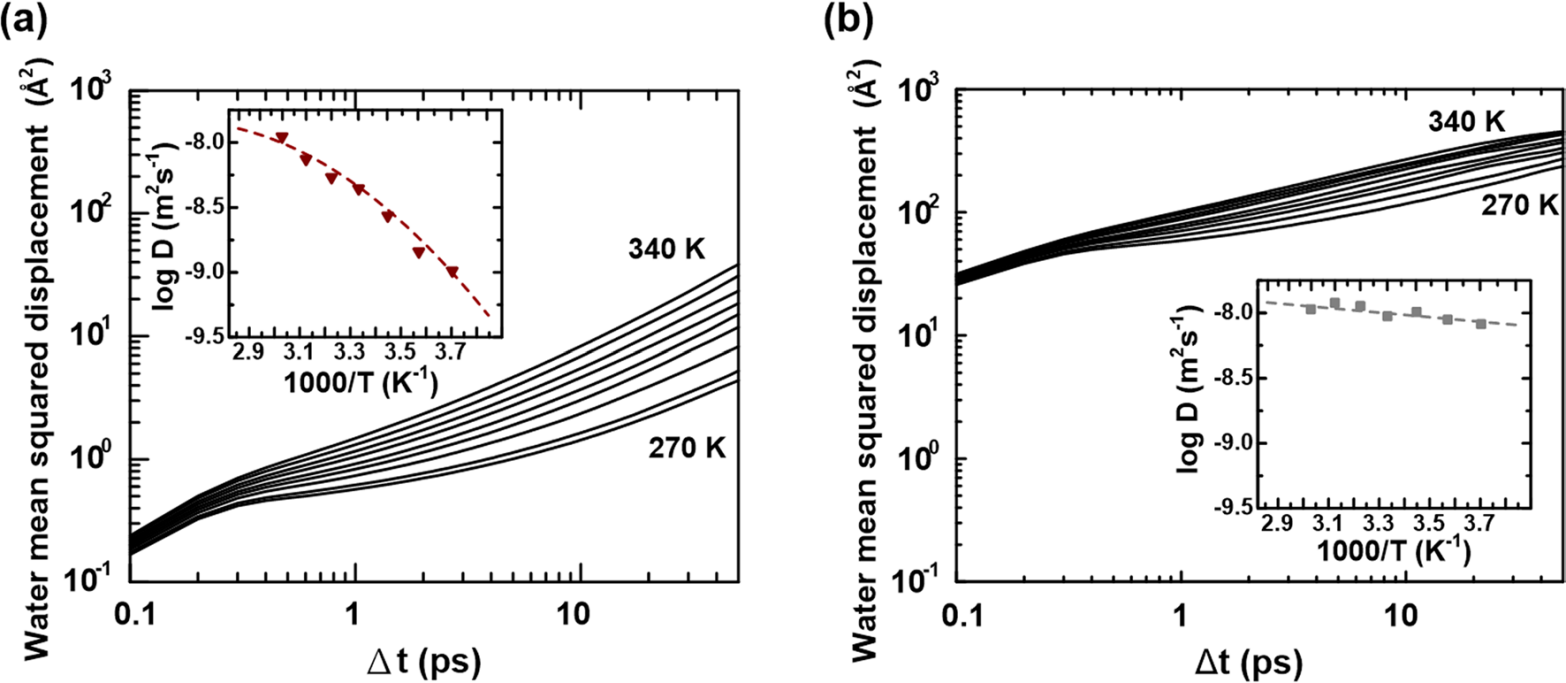
Statics of water MSD as a function of deviation time ∆*t* for (**a**) the interface channel waters and (**b**) the pure water system, respectively, accounting the temperature range of 270–340 K. The corresponding inset figures show the derived diffusion coefficients *D* as a function of the inverse temperature in the logarithm scale, 1*/T*.

We also carried out MD simulations to obtain the water diffusion dynamics in those three types of folded channels in the tetramer PFK-1. The mean squared displacement (MSD), Eq. (3), is measured over time to determine if water molecules are spreading solely due to diffusion. Single-particle tracking (SPT) enabled us to resolve the modes of motion of the diffusion of individual water molecules^50^. In the MD ensemble average, the time dependence of *MSD* for water motion in pure water is quite different from those of PFK-1 enzyme waters. Comparing Fig. 5a and Fig. 5b shows the dissimilar water motion behaviors of the enzyme-confined water and that of pure water without enzyme solute. As a result, the self-water diffusion coefficients *D* can be derived readily by Eq. (4)^50^. The self-diffusion coefficients *D* as a function of temperature are plotted in the corresponding figure inset, revealing an apparent difference in temperature-dependent water diffusion kinetics between the channel waters and pure water without the enzyme-confined matrix. The value of *D* for the channel water varies significantly with temperature in a non-monotonic way, while the pure water follows a monotonic linear change with temperature. The temperature dependence of channel water diffusion is described by Eq. (7), where the logarithm of estimated *D* against the reciprocal of the accessible temperature turns out to be a curve, rather than the straight line expected according to the Arrhenius law, Eq. (6).

We say that the reaction abides by a super-Arrhenius dependence, being associated with a convex curve, where plots are accounted for by an additional quadratic term in *1/RT* and the parameters elaborated in Table 1. For simplicity, we first discuss the results in terms of their activation energy *E*_*a*_, which is no longer a constant but depends on *1/T*, as compared with the cellular reparation barrier observation (Table 1). The trapped-water motion barrier does not remain constant, instead depending on the temperature as well as the channel confinement to which it belongs. From comparing these parameters and derived barriers, we observe that at low temperature, water molecules diffuse by crossing appreciable potential barriers, whereas at high temperature, the diffusive motions are barrier-less; the transient channel water motions seem significantly sensitive to ambient temperature, revealing 31.5 kJ mol^−1^ by 290 K and dropping to 23.0 kJ mol^−1^ by 310 K. These are in contrast to those of porous and interface water motions, which are revealed to be more steadily confined to a barrier region. The data analysis shows that in PFK-1 relevant water dynamics, the motion barriers are not just cumulative as respiration barriers; they primarily share a feature similar to super-Arrhenius behavior.

**Table 1:** Comparison of the derived super-Arrhenius parameters between PFK-1 water matrix dynamics and experimental respiration rates.

Type of water	PFK-1 water matrix dynamics with super-Arrhenius parameters^a^						
	ln D_0_	B (J mol^−1^)	C (J^2^ mol^−2^)	R^2^	E_a_ (290 K)	E_a_ (300 K)	E_a_ (310 K)
Transient channel	− 33.77	9.98 × 10^4^	− 1.58 × 10^8^	0.9998	31.5	27.1	23.0
Interface channel	− 19.90	2.49 × 10^4^	− 6.40 × 10^7^	0.9997	28.1	26.4	24.7
Porous channel	− 17.61	8.31 × 10^3^	− 3.86 × 10^7^	0.9999	23.7	22.7	21.7

	Respiration rate with super-Arrhenius parameters^b^						
	ln A	B (kJ mol^−1^)	C (kJ^2^ mol^−2^)	R^2^	E_a_ (290 K)	E_a_ (300 K)	E_a_ (310 K)
Experiment	− 155.42	9.47 × 10^5^	− 1.23 × 10^9^	0.9973	72.2	38.2	6.4

^a^Values of the parameters according to $\ln D = \ln D_{0} + B/RT + C/\left( {\left. {RT} \right)} \right.^{2} ,$ where *R* is 8.314 JK^−1^ mol^−1^ and the unit of *B* is J mol^−1^ and that of *C* is J^2^ mol^−2^ by temperature evaluation at 260–340 K, where temperature-dependent *E*_*a*_ values were estimated by Eq. (8) in units of kJ mol^−1^.

^b^Experimental data obtained from Ref.^8^, by refitting the measured data (see Fig. 1) to $\ln k = \ln A + B/RT + C/\left( {\left. {RT} \right)} \right.^{2} ,$ then deriving those parameters and temperature-dependent respiration barriers.

**Figure 6:**
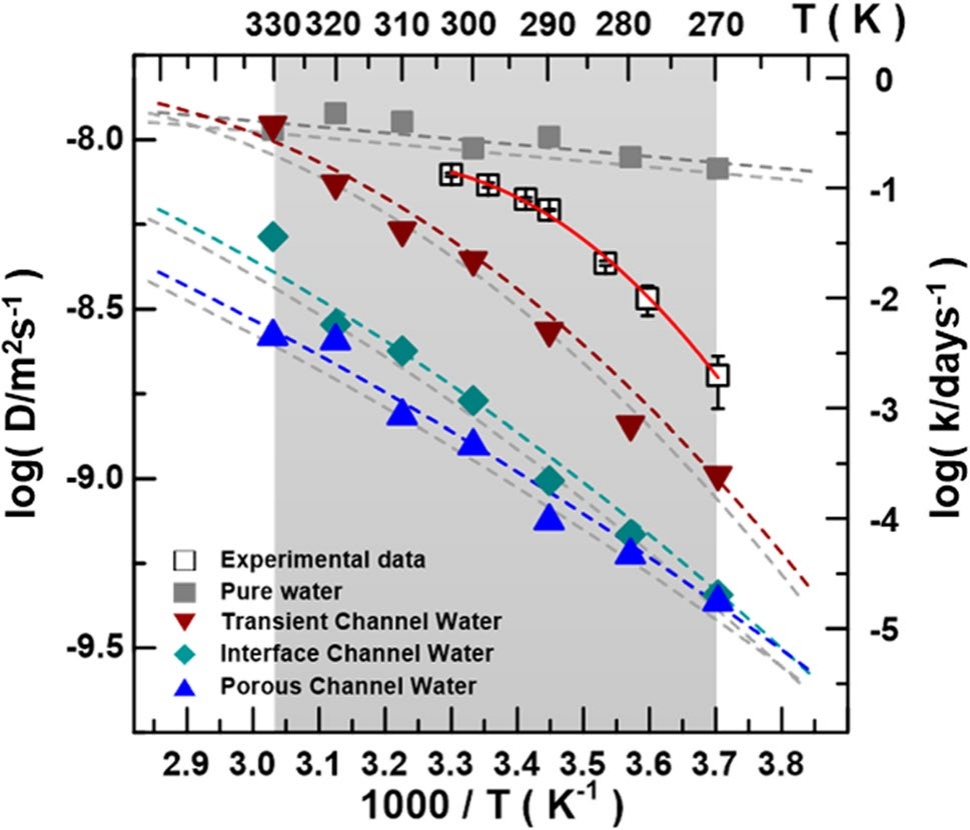
The temperature-dependent diffusion constant *D* of the pure water of the MD simulation box (gray square), the transient channel water (inverted red triangle), the interface channel water (green diamond), and the porous channel water (blue triangle) in the PFK-1 tetramer structure, respectively. The *D* unit is presented on the left vertical axis in the logarithmic unit, and the respiration rates *k* obtained from the experiment are plotted with the black hollow squares for comparison; the unit is given on the right vertical axis and the gray region is the temperature range of the MD simulation.

The water self-diffusion coefficient is directly associated with the tracer mutual diffusion of conjunction. Therefore, it is a fundamental transport property, essential for an accurate description of mass transfer relevant in the cellular respiration process, which is environmentally related^51^. This implies that respiration reactions are dominated by the entropy term because of the required water vehicle in facilitating the proton coupled electron transfer. Figure 6 presents a comparison of the temperature-dependent diffusion plots of the pure water and those for the transient channel water, the interface channel water, and the porous channel water of the PFK-1 tetramer structure, respectively. The axis of the reciprocal temperature *T* is presented at the bottom varying in the temperature range from 270 to 330 K. The temperature dependence for the bulk water diffusion looks almost monotonic against *1/T*, and the value of the diffusion coefficients for the pure water by temperature is ca. 8.2 × 10^–9^–1.1 × 10^–8^ m^2^ s^−1^ as a monotonic linear dependence. Compared with the channel water diffusions of the PFK-1 matrix, it clearly shows the temperature-dependent water kinetics in the significant super-Arrhenius behavior, with a primary similarity to that of the respiration rates *k* experimentally observed in the profile^8^. Moreover, Fig. 6 shows that increasing the temperature makes the channel waters move much more slowly than the tenfold magnitude of the diffusion coefficient quantity from that of the non-enzyme-confined pure water. Knowing the diffusion, we can also compare all the quantities of interest, such as the modulus of the diffusion coefficient. The results also show that the self-diffusion coefficients for the interface channel water at temperatures of 270–330 K change from 1.0 × 10^–9^ to 1.1 × 10^–8^ m^2^ s^−1^ for the transient channel, 4.6 × 10^–10^ to 5.2 × 10^–9^ m^2^ s^−1^ for the interface channel water, and 4.4 × 10^–10^ to 2.6 × 10^–9^ m^2^ s^−1^ for the porous channel.

Figure 6 reveals the case of a markedly convex curve feature in the nano-channel confinements as compared to that of pure water. These super-Arrhenius-behavior-like profiles exhibit the constraint confinement to microscopic interpretations^12^. For such a PFK tetramer complex, we suppose that the reaction configuration space may be partitioned into two types of conformations, one of which (called the open state) is reactive, with a diffusion coefficient *D(T)*, and the other of which (called the closed state) is nonreactive. This provides a mechanism for *D(T)* to decrease with *T*; i.e., the water density of the state decreases with *T* faster than *D(T)* increases, see Fig. 4(b). This could occur because higher-diffusion water molecules visit a wider region of the phase space and hence spend a smaller fraction of their time in the constrained region, referring to the transient channel water behavior in Fig. 4(b) and Fig. 6. Such more sensitive water number variation in the transient channel by temperature is reflected in its diffusion rate. The more limited transient channel spaces compared to those of the interface channel and porous channel (see Fig. 4b) lend the corresponding water dynamics an apparent feature of super-Arrhenius behavior in Fig. 6. This interpretation may be of significant importance for enzymology. In recent years, several studies have focused on understanding the role of conformational sampling in enzyme catalysis^12^. There is still room for arguing that the active configurations sampled by PFK-1 enzymatic complex are not of higher energy than the general population of conformers and that populating these conformations of specific water/substrate channel network may determine the reaction rate^38^.

Under a certain temperature range, the long-term slowing of self-diffusivity is featuring a super-Arrhenius trend. Our results suggest smaller channel spacings the continuous changing of energy barriers due to the continuous variation of water configurational excess entropy with temperature. It appears that the Arrhenius to super-Arrhenius behavior could be a more generic feature arising from the interplay of water confinement motions and temperature effects rather than the details of the chemical reaction steps^15^. Note also that such a scenario agrees with the findings of Ref.^14^, where it is demonstrated that the Arrhenius to super-Arrhenius crossover takes place for smaller spacing confinement of argon gas diffusion at lower temperatures. We discuss the significance of this observation, and we provide an interpretation of this finding by combining experiments and simulations. The accuracy of these simulated water diffusion coefficient quantities is considered and compared to the earlier experimental measurements^22,52,53^ in Table 2, since data for water diffusion coefficients over temperature in multiple condition systems have been reported for pure water, isotope water, nanocarbon tube water, and aquaporin’s channel water, respectively^22,52,53^. The most significant difference seems to be that for the maximum water diffusion coefficient appearing in bulk water. Moreover, our results are consistent with the measurements for systems that show maxima at about 10^–8^ m^2^ s^−1^, depending on the examined temperature, and provide evidence of the sensitivity to the temperature effect of protein matrix channel bio-water diffusion.

**Table 2:** Self-diffusion constants of different confinement water dynamics by temperature.

Systems (simulation/experimental conditions)	Channel			Diffusion coefficients (10^–9^ m^2^/s)	Activation energy, E_a_ (kJ/mol)	References
	diameter (Å)	Length (Å)	Water			
Pure water	–	–	–	8.24–10.64	7.03	This work
PFK-1 interface channel^a^	6.0 ± 1.5	85.9 ± 8.5	16–28	0.30–1.88	18.37	This work
PFK-1 porous channel^a^	5.9 ± 1.5	91.4 ± 3.5	4–7	0.28–1.74	21.92	This work
PFK-1 transient channel^a^	5.2 ± 1.7	54.7 ± 17.5	3–17	0.37–1.94	32.58	This work
Pure water^b^	–	–	–	1.00–4.16	4.58	^53^
Millipore water filter^c^	–	–	–	1.30–2.74	17.8	^52^
Aquaporins^a,d^	4.5 ± 1.7	~ 50	8	0.27–0.69	31 ± 3	^22^
Aquaporins treated^a,d^	4.5 ± 1.7	~ 50	8	0.18–0.51	40 ± 3	^22^

^a^The channels were computed using MOLE*online*^46^ for PFK-1 (PDB entry 1PFK) and Aquaporins (PDB entry 2ABM), excluding ligands, and *E*_*a*_ was calculated at 290 K.

^b^The self-diffusion coefficients of liquid water in the temperature range 273–325 K were determined by both the capillary method and the usual diaphragm cell method with deuterium as tracer.

^c^Experimental data are from the self-diffusion coefficients of a millipore filter diaphragm cell of water in the temperature range 278.2–308.2 K.

^d^The Arrhenius plots of the logarithm of D_ef_ versus *1/T* of both control and HgCl_2_-treated roots had linear portions between 293 and 283 K and between 298 and 308 K. *E*_*a*_ values obtained from the slopes of linear portions of the graphs differed at 293–303 K.

As manifested above, the self-diffusion of water in the bulk phase and under confinement, which is very relevant to characterizing the dynamic bottlenecks of the water-utilizing allosteric control between multiple effector and active sites in PFK-1 tetramer. We demonstrate that the water density of the state changes by temperature, quantitatively describing the accumulation network caught in between the active and effector sites. As the temperature increases, water molecules have higher kinetic energy to escape the channel confinement; from channel water numbers estimated by temperature (Fig. 4b), we present three aspects—residue distribution, trapped water number, and time trajectory variation—to distinguish the channel water behaviors. The residue distribution and the total water number are complementary, for in the interface channel, the hydrophilic residues are evenly distributed, which leads to the smaller water number and causes the water network to form a two- or even one-dimensional water network. In the long porous channels, more hydrophobic residues are occupied and cause more water molecules to be trapped, inhibiting the hopping or transfer along a one-dimensional water chain alignment. In the transient channel, the good distribution of alternative hydrophilic and hydrophobic residues keeps the water number appropriate, and the one-dimensional water chain may help the communication of sites. For example, two water molecules suspended in a narrow channel tend to orient in one-dimensional contact because of the water alignment induced by the hydrogen bonding network.

Our study recognizes that as the temperature rises, thermal energies will become larger than barrier heights^20^. What is required for a super-Arrhenius plot is that the average energy of water molecules making transitions by diffusive hops between sites along the channel rises more slowly than the average energy of those bulk (or pure) water molecules. This is due to the water diffusion kinetics in the PFK-1 tetramer matrix, wherein the water molecules interact strongly with the protein amino residues; those bound water molecules in the protein surface and in the interior channels, which occupy internal cavities and deep clefts, interact with the amino residues with different chemical characteristics and are extensively involved in the water hydration H-bonding, in conjunction with potential sites, and thus exhibit assorted dynamics.

Nevertheless, the respiration process with water transport mentioned above may be complicated by multiple parallel pathways in cellular organisms. This study highlights water motions in the PFK-1 tetramer complexed channel, which have yielded to detailed analysis compared to those water-related systems. The super-Arrhenius behavior manifests the basic features of PFK-1 channel water dynamics in collective phenomena such as enzymatic catalysis-promoted respiration processes. The temperature dependence of rates of enzymatic catalysis reactions has been identified, such as the super-Arrhenius kinetics, where transport phenomena accelerate as the temperature increases^17^. Although such an examination is expedient and appropriate for the complicated respiration processes, the entropy of the activation term should never be overlooked because some reactions, in particular those involving strongly-solvated ions, may be dominated by entropy. As illustrated in Fig. 2, which presents the magnesium ion coordination, this domination is due to water solvation effects.

It appears that one can acquire a more fundamental understanding of the very complicated respiration processes by applying the super-Arrhenius kinetic and reaction modeling on a molecular level. An important remark regarding the MD simulations is that these dynamics of structural modulation involve a locally-constrained environment of protein and the trapped-water motion within the channel^54^. The trapped-water motion takes place in different ways, and our interest lies in how such water molecules in the active or effector site could be regulated in different channels with different structural states in terms of water diffusion in the channels. It seems likely that the hydrophilic nature of the active/effector site and channel make it favorable for water molecules to remain inside, and they can move in and out of these sites through the channels and those interfaces, for the channel structures are dynamical. A water network in conjunction with potential sites between the crystallographic structure and the MD simulation is thus conceivable. Presumably, the channel water might also be associated with a water hydration network in precise control of the communication of different sites and conformational change.

Therefore, we conclude that channel water diffusion must play a key role in controlling the respiration reaction of the Camellia plant, assuming that the enzymatic catalyzed reaction at PFK-1 is the rate-determining step in glycolysis. This result on channel water diffusion also suggests that the interaction of water in the channels changes due to a stronger perturbation of seclusion by binding sites associated with the channel wall conformation in the network, and such interaction would vary with temperature. Accordingly, we expect that the present MD simulation clarifies the key point that the temperature dependence of the water diffusion in the channels of PFK-1 should be able to provide us an important clue about the interconnection mechanism with water molecules and how they exchange controlling signals between allosteric sites of the PFK-1 tetramer matrix in the cellular respiration reaction. We provide an interpretation of this finding that places it in the broader perspective of water transport as well as biochemical kinetics.

## Summary

We report in this work the temperature dependence of channel water diffusion in the PFK-1 tetramer matrix, determined by MD simulations, thereby providing a molecular understanding of the water transport role in the super-Arrhenius behavior of the respiration rate of the Camellia plant obtained in our previous experiment. We found two stable types of water channels of PFK-1, classified as (1) the interface channel and (2) the porous channel, and a third water channel, the so-called (3) transient channel, which is structurally rather less stable. These channels serve as interconnecting pathways with confined water diffusion in channels between active sites and effector sites of the enzyme, as well as linking to the outside bulk water. The temperature dependence of PFK-1 channel water diffusion is revealed by super-Arrhenius dependence, being associated with a convex curve, and the activation energy *E*_*a*_ is no longer a constant but depends on *1/T*, which suggests a surprising similarity to the cellular reparation barrier observation. Our results suggest that the water molecules in the PFK-1 tetramer matrix protein surface and in the interior channels are extensively involved in the water hydration H-bonding in conjunction with potential effector and active sites and thus exhibit the assorted dynamics. Such super-Arrhenius temperature dependence of the channel water diffusion provides the important information that the water in the scheme of the respiration reaction, proposed as C_6_H_12_O_6_ + 6H_2_O + 6O_2_ → 6CO_2_ + 12H_2_O, can be interpreted as a transport medium linking allosteric sites of the PFK-1 enzyme through diffusion in the channels, but not as a simple reactant molecule, as usually understood. It is thus important to point out that we should be careful in clarifying the role of water, depending upon its location in the enzyme system. The present MD simulation results demonstrate that a comparative study provides insight for further clarification of the water-utilizing allosteric control between multiple effector and active sites in enzymes.

## Methods

### Molecular dynamics simulation

Because the PFK-1 A subunit has more similar reaction conformations than the B subunit, molecular dynamics simulations were performed on a globular protein constructed of a PFK-1 (PDB entry 1PFK) A subunit, referred to as a many species structure^1,15,45,55,56^, in of dilute aqueous solution. MD simulations were performed with the AMBER 14 package^57^ using a 0.1 ps integration time step and periodic boundary conditions with the 124 × 115 × 131 Å^3^ box. The proteins were modeled with the Amber ff14SB force field^58^ and solvated with the TIP4P-Ew water model, and a set of GAFF^59^ parameters was adopted for the description of fructose-1,6-bisphosphate (F-1,6-bP) and fructose-6-phosphate (F-6-P). The fully solvated and ionized system contained 238 thousand atoms with about 56,000 TIP4P water molecules.

For the optimization cycles (3000 steps for the steepest descent and total max 20,000 steps for the conjugate gradient), the system density and box lengths were converged to reach equilibration; the system density was stabilized at ~ 1.06 g/cm^3^. Then the system underwent the each 1 ns annealing NPT ensemble with equilibrated steps from 0 to 340 K, which temperature gap is 10 K, at every target temperature and a target pressure of 1.0 bar at a collision frequency of 1 ps^−1^ by using a Langevin-thermostat. Form 0 to 150 K do the onion restraint with the center of active site by the force of 500 kcal mol^−1^ Å^2^ and release every 2 Å form the outer to the active site residues at every 10 K till release all the residues. The MD sampling of temperature 260—340 K is during the heating and density dynamics and further equilibrated the systems for 10 ns to get well-settled pressure and temperature for conformational, chemical analyses and take the final 300 ps of the trajectory for the RMSD and diffusion calculation. All structural analyses were performed with Visual Molecular Dynamics (VMD)^60^.

### Channel search and channel classification

The channels in PFK-1 were computed in MOLE*online*^46^. Any cavities were identified and their start and end points linked. Various channel properties and scale were computed and tabulated according to the amino acids. In the submitted version, we count the amount of water at around 5 Å of the channel amino acid under cumulative time. At the prompting of the reviewer, we noticed that this definition counted more water molecules trapped inside the protein and interfered with our observations, so we adjusted the statistical definition and calculated the number of water molecules at 3 Å around the center of the channel. The result is shown on the right side of Fig. 4b.

We employed MOLE*online* software^46^ to search for the channels of the PFK-1 tetramer enzyme from the protein data bank with Phosphofructokinase-1 (PDB entry 1PFK)^45^. Starting points for the channel search were set at the center of the enzyme, and also at four active sites and four effector sites of PFK-1, to clarify the channels (or tunnels) that interconnect possible buried protein cavities within the protein surface. The probe radius was set at 20 Å. The current version of MOLE*online* uses the Dijkstra’s Shortest Path Algorithm to find the channels between starting and ending points, and also employs Voronoi diagrams for constructing the approximated structure, in which individual atoms are replaced by 4, 8, 12 or 20 spheres with a radius of the smallest atom of a given protein of PFK-1^46^. Under this approximation, the Voronoi diagram made it possible to construct correct channel structures and identify water paths. By algorithm search and careful examination, we are able to classify the channels into three types, one less-stable transient channel and two constantly stable channels, namely, the interface and the porous channels, as shown in Fig. 3.

### Water diffusion coefficient calculation

To distinguish whether the dispersion behavior of particles is caused by diffusion, it can be determined by the mean-squared displacement (MSD) ,and using Langevin equation to try to describe the diffusion of Brown particles^3^, where *MSD* is defined as

3 $$ {\text{MSD}} = \left( {x - x_{0} } \right)^{2} = \frac{1}{N}\mathop \sum \limits_{n = 1}^{N} \left( {x_{n} \left( t \right) - x_{n} \left( 0 \right)} \right)^{2} $$

where *N* is the number of particles to be averaged, $x_{n} \left( 0 \right) = x_{0}$ is the reference position of each particle, and $x_{n} \left( t \right)$ is the position of each particle in the determined time t. Thus, the directed motion of diffusion is given by Eq. (3).

4 $$ r^{2} = 4Dt + \left( {Vt} \right)^{2} $$

where $r^{2}$ is the mean-square displacement, *D* is the simultaneous diffusion, *t* is the time, and *V* is velocity.

5 $$ {\text{MSD}} = x^{2} = 4Dt + \left( {Vt} \right)^{2} $$

By comparing Eqs. (4) and (5)^50^, we obtain the *MSD*, which can be used to derive the water diffusion coefficients as a function of time in the logarithmic scale in *ps* for pure water without the enzyme as the reference, the corresponding channel waters. Then the corresponding *D* can be plotted as the function of the inverse temperature in the logarithm scale, 1*/T*, respectively.

### Approach to temperature dependence of water diffusion kinetics

The more literature refers that the temperature-dependent rate is to consider deviations from the Arrhenius law so that it can more accurately simulate the diverse phenomena that modern chemical kinetics need to solve. It is outlined how such deviations manifest as concave or convex Arrhenius plots and can be referred to as sub- and super-Arrhenius behavior, respectively. In Fig. 2, the logarithm of the observed respiration rate against the reciprocal of the absolute temperature *T* seems to deviate from a straight line, as expected according to the Arrhenius law,

6 $$ k\left( T \right) = A \exp \left( { - \frac{{E_{a} }}{RT}} \right)\quad {\text{or}}\quad \ln k\left( T \right) = \ln A - \frac{{E_{a} }}{RT} $$

where *A* is the pre-exponential factor and *E*_*a*_ as the activation energy. *E*_*a*_ is assumed to be independent of temperature in Eq. (6). However, in most situations of solution biochemistry interest, the accessible temperature rate measurement turns out to be a curve, rather than the straight line expected according to the Arrhenius law^13^. In Ref.^8^, deviations from linearity in an Arrhenius plot are accounted for by an additional quadratic term in *1/RT* according to Eq. (7).

7 $$ \ln k\left( T \right) = \ln A + \frac{B}{RT} + \frac{C}{{\left( {RT} \right)^{2} }} $$

where *C* dictates whether the plot is ‘concave’ or ‘convex’^12,13,18^, and the reaction abides by a sub- or super-Arrhenius dependence, such that the activation energy *E*_*a*_, being connected to the slope of the Arrhenius plot, as Eq. (6) shows, is no longer a constant but depends linearly on *1/T*, by Eq. (8).

8 $$ E_{a} \left( T \right) = - \frac{d \ln k}{{d \left( \frac{1}{RT} \right)}} = - B - \frac{2C}{{RT}} $$

here the *C* coefficient, which is given by Eq. (9), is negative or positive for the sub- or super-Arrhenius cases, respectively.

9 $$ C = \frac{1}{2}\frac{{d^{2} \ln k}}{{d \left( \frac{1}{RT} \right)^{2} }} $$

Aquilanti et al. provide a molecular level examination to endorse the reciprocal-activation-reciprocal-temperature relationship^12,17^, by extending the Tolman theorem^12,17^, and following the interpretation by Truhlar et. al.^12,17^. The function *Ea*, is written as the logarithmic derivative of the rate constants with respect to *1/RT* (Eq. 8). The specific transitivity can be interpreted by defining the reciprocal as a measure of the propensity of the reaction progress^12,17^ to proceed the process. Since we demonstrate a description of the rate departures upon temperature to the McLaurin and Taylor series, considered as a differential equation for *k(1/RT)*, is of first order in the variable (*1/RT*) and is easily integrated, specifying the lower limit of the integration range, yielding the deformed Arrhenius. This can be described from the thermodynamic limit as due to the interruption of a discrete temporal sequence of events^12,17^. One can understand this considering that to accumulate the respiration reaction process needs a sequence of favorable collisions and particle diffusion transports.

Let’s elaborate further on the extension to the deformed exponential rate equation (Eq. 7) for the connection between super-Arrhenius and water fluid diffusion kinetics, which adequately describes the relationship between the diffusion coefficient and temperature. In general, for particles in an ideal gas the diffusion coefficient can be predicted from first principles by taking the values for the mean free path and average velocity from the Maxwell–Boltzmann distribution; for molecules in a viscous fluid (or a liquid solution), they obey Stokes–Einstein equation. Note that for solvent molecules under a specific confinement the viscosity itself strongly depends on temperature, and the diffusion coefficient *D(T)* normally obeys the relevant Arrhenius relation, Eq. (10)

10 $$ D\left( T \right) = D_{0} \exp \left( { - \frac{{E_{a} }}{RT}} \right) $$

Here, *Ea* is an "activation energy of diffusion"; in a super Arrhenius-like case assume that diffusion coefficients in the solution phase grow faster than exponentally with temperature and a law Eq. (11)

11 $$ \ln D\left( T \right) = \ln D_{0} + \frac{B}{RT} + \frac{C}{{\left( {RT} \right)^{2} }},\quad E_{a} \left( T \right) = - \frac{d \ln D}{{d \left( \frac{1}{RT} \right)}} = - B - \frac{2C}{{RT}} $$

similar to Eq. (7): the fact is here emphasized that the transmissibility may not be constant, and the temperature-dependent diffusion coefficient *D(T)* can be described according to a deformed Arrhenius type of formula, so that low order derivatives of $\ln D$ with respect to 1*/RT* can be extracted by molecular dynamics simulation and compare to experiments when available^12,17–19^. Thus the parameters measure deviations between equilibrium and non-equilibrium situations leading to the continuous generalization of the diffusion states as series of replicas of microscopic ‘entities’, the specific configurations depending on temperature, The coefficient *B* and *C* account for transport behavior attributed to the occurrence of long series of events. Collective phenomena such as the rate of the enzymatic catalysis process, food preservation process, and the basic features of the dynamics of complex or glass-forming liquids and solids have exhibited Super Arrhenius behavior^12,17–19^: a significant number of studies on the temperature dependence of rates of biophysical reactions have been analyzed and found to manifest the super-Arrhenius kinetics, where transport phenomena accelerate as the temperature increases faster than according to the Arrhenius law. The role of transitivity^12,17–19^ is a promising target of further studies.
